# A Case of Spontaneous Gangrene of Right Lower Extremity

**Published:** 1859-09

**Authors:** George Schloetzer

**Affiliations:** Chicago


### ARTICLE III.

### A CASE OF SPONTANEOUS GANGRENE OF RIGHT LOWER EXTREMITY.

REPORTED BY GEORGE SCHLOETZER, M.D., OF CHICAGO.

Mrs. S., aged 43 years, was attacked, in the early part of February last, with an apparently slight indisposition, accompanied with a little œdema of the upper lids, which was attributed to an erysipelas eruption, from which the patient had frequently suffered. Except during these attacks of erysipelas faciei and an attack of typhus fever four or five years ago, she states that she had always enjoyed good health. Menstruation has always been regular. Patient has borne five children, each labor having been attended with considerable difficulty.

On the 6th and 7th of February, she experienced a peculiar sensation in the toes, and shortly afterwards, pain in the region of the hip and right side of the pelvis, which extended downwards as in sciatica. Mrs. S. supposed these pains proceeded from rheumatism, and as they continued to increase in severity, she called her physician on the 8th, who also at first regarded the disease as rheumatism. As there was some fever, he prescribed antiphlogistic remedies, administered pot. nit., etc., and ordered cups and blisters. Soon after, other important symptoms supervened. The toes and then the whole limb became very cold; sensation and motion were soon lost; the toes gradually assumed a gangrenous aspect; the skin of the leg became livid red; and large, bluish spots soon appeared upon the thigh. On the 9th, at the regular period, the menses commenced, and continued three or four days.

On the 12th of February I was called in consultation. I found the distal half of the toes black and dry (mummified), as we often see in gangræna senilis; the leg, and nearly the whole of the thigh, was covered with large, dark, violet-red maculæ; below the knee, the limb was without sensation and power of motion; the foot was cold, and the natural heat of the thigh was much diminished, except at the uppermost, where it was normal; the right popliteal and femoral arteries were without pulsation; the pulsation of the left femoral artery was stronger than in health; the rheumatic pains, which were now more like those of neuralgia, were still present, but somewhat diminished in violence, and resembling formication. Pulse 115. Upon examination of the heart, I could find nothing abnormal; in the lungs, however, were extensive rales, accompanied with cough; tongue coated; appetite small; bowels confined.

Upon a careful examination, I was disposed to refer all the symptoms to arterial obstruction; whether produced by arteritis, thrombosis, or by pseudo-plasma (infiltration, tumor), in the region of the large vessels, must remain doubtful; probably, however, the obstruction was caused by thrombosis—embolic (coagulum formed in a larger vessel and carried by the blood to the opening of a smaller one).

The prognosis was certainly very unfavorable as regards the limb, with scarcely a hope of saving life. It was now determined, since antiphlogistic remedies had already been employed, to give those of a supporting and stimulating character—cinchona decoct., quinia, wine, nutritious beef tea. Particular regard to change of air was enjoined. Heated woolen coverings were placed around the limb, which was repeatedly rubbed with spirituous lotions and turpentine. On the next day, electricity was applied to the limb and continued several days. Heated sacks of sand were also placed around the leg and thigh, by means of which a considerable degree of artificial heat was retained. No pulsation reappeared in the arteries, and no remedy seemed to exert the least influence upon the course of the disease. Still, on the 16th of February, the radial pulse seemed to improve, being 90–95, and of better quality.

The integument of the whole limb became more firm, dry and parchment like, and of a darker color. During these modifications, however, the toes scarcely changed their appearance. The stools, which at first had been somewhat confined, were afterwards (Feb. 20th) more frequent (from two to four daily) and watery.

During the evening and night the fever was more violent. The patient had become much emaciated; the pain in the limb was now slight, unless it was moved, or pressure applied to the thigh, near the line of demarkation.

*Feb. 22nd.* The healthy and unhealthy tissues have begun to separate. Subcutaneous emphysema, from generation of gas, had already pervaded the upper portions of the thigh. Except near the line of demarkation, the dry gangrenous process was confined to the external tissues, and the moist to the internal.

The question now arose whether it was advisable to remove the diseased portion by amputation. In consultation with several physicians, it was decided not to operate at present, since rales were heard throughout the whole chest, since dullness on percussion existed in the abdomen, which seemed to indicate an exudation into the peritoneal cavity, and since, further, the sloughing extended, on the internal aspect of the thigh, nearly to the right labium, and on the external aspect, almost as high. Moreover, there continued a feverish pulse; diarrhœa. The strength was failing, and emaciation becoming extreme.

*March 1st.* The space formed by the separation of the unhealthy from the healthy tissues is becoming broader and deeper. A fetid discharge flows from the fissure; in other parts are formed phlyctenae and sloughs, from which exudes the same kind of fetid discharge. The size of the limb is not much increased, since the generated gases and fluids pass off through the sloughs and small incisions which have been made for the purpose. The left foot and leg is already becoming quite œdematous. Pulse feverish (115–125) and soft.

*March 5th.* There has been for several days a remarkably strong pulsation of the abdominal aorta. Tongue dry, and bright red, as if deprived of its epithelium; no appetite; fluids can only be swallowed with difficulty; there is great restlessness; a colliquative diarrhœa is rapidly reducing the patient's strength.

*March 7th.* Patient has succumbed. During the last few days there has been no marked change in the general appearance of the limb. The soft tissues, including the muscles, arteries and nerves, upon the inner and posterior aspect of the thigh, embracing nearly two-thirds of its circumference, are destroyed almost to the bone. No autopsy was permitted.

[We append to the foregoing case some notes by Prof. Blackman on the subject of gangrene, which will be found interesting. They are from the *Cincinnati Lancet and Observer* for 1859.]

Cases of cerebral gangrene, resulting from obstruction of the cerebral vessels by detached "cardiac vegetations," have been reported by Kirkes, Ruhle, Tuffnell, Burrows and Virchow. Mr. Paget has also collected a number of cases in which pulmonary gangrene was attributed to the same cause; but of the seventeen cases collected by him, none occurred in the puerperal state.

I confess when I first saw my patient I was not aware that mortification of the extremities had ever been observed in the puerperal state. Since my attention has been directed to the subject, however, I find that it has been discussed by Prof. Simpson, in the second series of his Obstetrical Memoirs; and by Virchow, in his *Gesammelte Abhandlungen*, etc., published at Frankfort in 1856. During the past six months, also, a series of papers by Charcot and Bawl has appeared in the *Gazette Hebdomadaire*, in which may be found an excellent summary of what has been recorded upon this subject. Dr. Benjamin Ward Richardson, also, in his recently published Astley Cooper Prize Essay on the cause of the coagulum of the blood, has particularly referred to the influence of the puerperal state in favoring the deposition of fibrin during life; and his remarks in reference to the matter under consideration are worthy of careful study.

There is abundant evidence to show that gangrene of the internal organs, or of the extremities, may be produced by the plugging of the arteries which supply them with blood; and the next question that presents itself is, what is the condition of the blood, or of the arterial coats, which favors the formation of the fibrinous deposits which thus occlude the vessels? Formerly nearly every case of spontaneous gangrene was referred to a fatty or osseous degeneration of the arterial coats, and such mortification was supposed to be peculiar to old age. At length, cases were reported occurring even in children, and we suppose that most physicians, who have been long engaged in practice, have met with the disease in persons under fifty. Dupuytren and Syme have strongly maintained that it is due in all cases to arteritis, an inflammation of the lining coat of the artery. Now the experiments of Virchow, and more lately of Mr. Henry Lee, of King's College Hospital, London, have proved that both chemical and mechanical irritants produce their effects only on the external and middle coats of arteries, and on the outer coats of the veins. It has been found to be a matter of great difficulty to produce, by experiment, an effusion of lymph on the non-vascular lining membrane of arteries; but when once the inner lining has been detached by violence, or removed by disease, the tendency is very strong towards the formation of fibrinous deposits, which may be lodged in any of the organs to which such artery may be distributed. Dr. Oke, of Southampton, England, has reported a case in which the brachial artery became suddenly obstructed after abortion, which obstruction he attributed to the spontaneous rupture of the inner coat of the vessel, and the projection of its edges into the tube. He bases his opinion upon the fact that no abnormal action of the heart could be detected when the obstruction in the left axillary artery took place, nor subsequently, the patient having recovered with the loss only of the integument of the thumb and fingers. Although this case is quoted by Professor Simpson, under the head of "Arterial Obstruction from Spontaneous Laceration, and Corrugation of the internal coat of the Arteries," I feel inclined to question this explanation of the case, for in my own patient Professor Lawson was unable to detect anything abnormal about the heart, and I believe I shall, in the course of my remarks, furnish a more rational view of its pathology. The general fact, that a detached inner coat does favor the obstruction of the artery, cannot be questioned, for it has been well established by experiment, and illustrated by disease.

